# Felty Syndrome Presented With Candida albicans Lung Abscess Without Arthritis: A Case Report

**DOI:** 10.7759/cureus.66989

**Published:** 2024-08-16

**Authors:** Mohammed Ahmed, Mohanad Abdelrahim, Mortada Mohammed, James Cyril

**Affiliations:** 1 Cardiology, Aintree University Hospitals NHS Foundation Trust, Liverpool, GBR; 2 Internal Medicine, St Helens and Knowsley Teaching Hospitals NHS Trust, Liverpool, GBR; 3 Internal Medicine, Metrohealth Medical Center, Case Western Reserve University, Cleveland, USA; 4 Internal Medicine, Wexford General Hospital, Wexford, IRL

**Keywords:** felty syndrome, rheumatoid arthritis, complication of rheumatoid arthritis, lung abscess, candida albicans

## Abstract

Felty syndrome (FS) is a late manifestation of severe active rheumatoid arthritis (RA). A high index of suspicion or FS is needed in patients who present with neutropaenia and splenomegaly with no initial or obvious identifiable cause. We present the case of a 52-year-old who presented with a one-week history of haemoptysis, fever, and night sweats. The patient was hypotensive, tachycardia, and febrile (38 °C). On examination, bilateral crackles and reduced air entry were identified on the right basal and middle zones. The patient was diagnosed with RA two years prior to this presentation and was not on a disease-modifying antirheumatic drug (DMARD). Haematology showed high inflammatory markers and pancytopenia. Chest X-ray showed a right upper lobe abscess. CT-thorax, abdomen, and pelvis confirmed lung abscesses and hepatosplenomegaly. Candida albicans was detected on the broncho-alveolar lavage. He responded well to antifungal medication and corticosteroids with normalisation of the pancytopenia and inflammatory markers and reduction of the spleen size. This case report details the unusual and early presentation of FS in a patient newly diagnosed with RA and who had no active arthritis. We wish to emphasize the importance of a high index of suspicion in patients with RA regardless of the length of their illness.

## Introduction

Felty syndrome (FS) is an uncommon extra-articular manifestation of seropositive rheumatoid arthritis (RA), which was first described by Augustus Felty in 1924 at Johns Hopkins Hospital. He described five unusual cases with common features of chronic arthritis of about four years duration, splenomegaly, and leukopenia. FS is characterised by a triad of chronic arthritis, splenomegaly, and neutropenia. Completion of the triad is not necessary for the diagnosis, but neutropenia is a hallmark feature of the disease and cannot be absent [1].

The prevalence of FS is approximately 1-3% among the RA population [2]. FS usually occurs in patients with severe, long-standing, erosive, and seropositive arthritis. However, the diagnosis of FS can precede arthritis symptoms in a few cases, and it is considered a cause of neutropenia even in the absence of joint symptoms [3].

The presence of neutropenia in monitoring blood tests in patients with RA could be the first presenting feature of FS as it is usually asymptomatic with dermatological and pulmonary infections reported as the most common presentations. Extra-articular manifestations have been reported in many patients, including rheumatoid nodules (74%), hepatomegaly (68%), lymphadenopathy (42%), Sjogren syndrome (48%), pulmonary fibrosis (50%), pleuritis (22%), peripheral neuropathy (14%), and leg ulcers (16%) [4].

Large granular lymphocyte (LGL) leukaemia must be considered in any suspected FS, and 30%-40% of FS cases may have LGLleukaemia expansions [5].

## Case presentation

We present a 52-year-old male diagnosed with FS after two years of initial diagnosis of seropositive RA, and the patient was not on disease-modifying antirheumatic drugs (DMARD) or any active treatment for RA for unclear reasons. The patient presented with a one-week history of haemoptysis, fever, and night sweats. The patient further reported weight loss of 6 kg over the last month. Systems reviews were unremarkable with no joint involvement. His past medical history was significant for basal cell carcinoma (BCC) and depression. The patient's only medication was sertraline. He was a heavy smoker, 30 packs/year, and reported heavy alcohol consumption.

**Figure 1:**
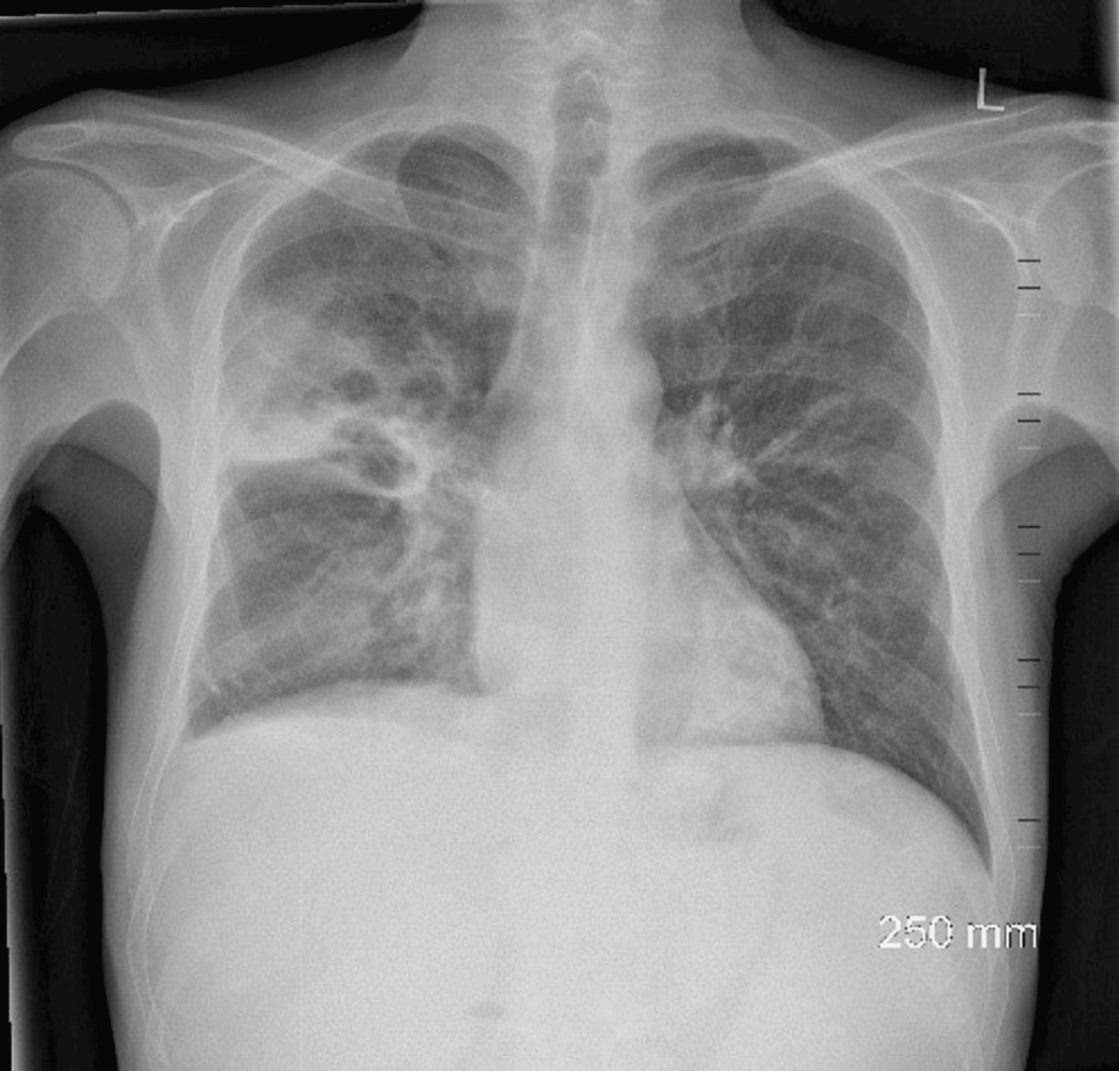
Chest X-ray on admission

**Figure 2:**
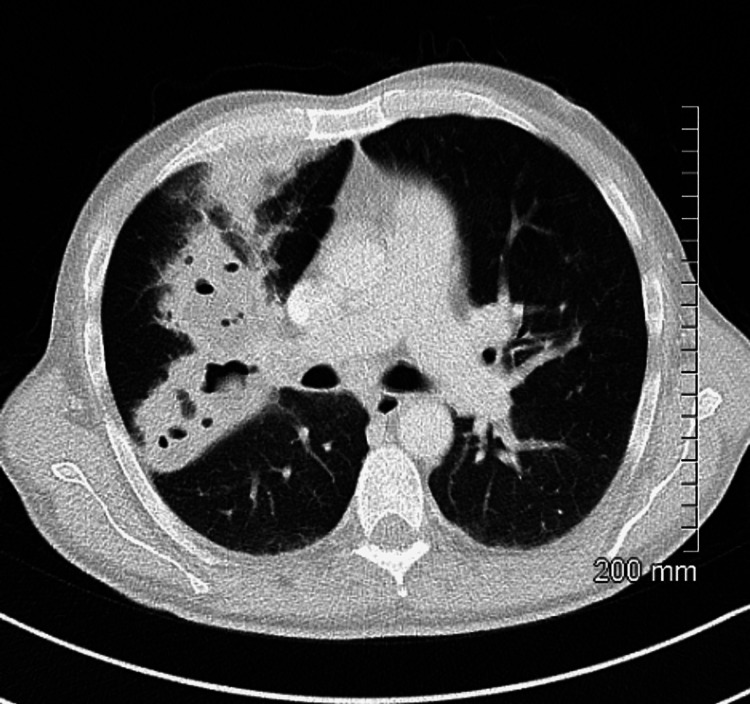
CT chest on admission showing the confluent intrapulmonary lesion suggestive of lung abscesses

**Figure 3:**
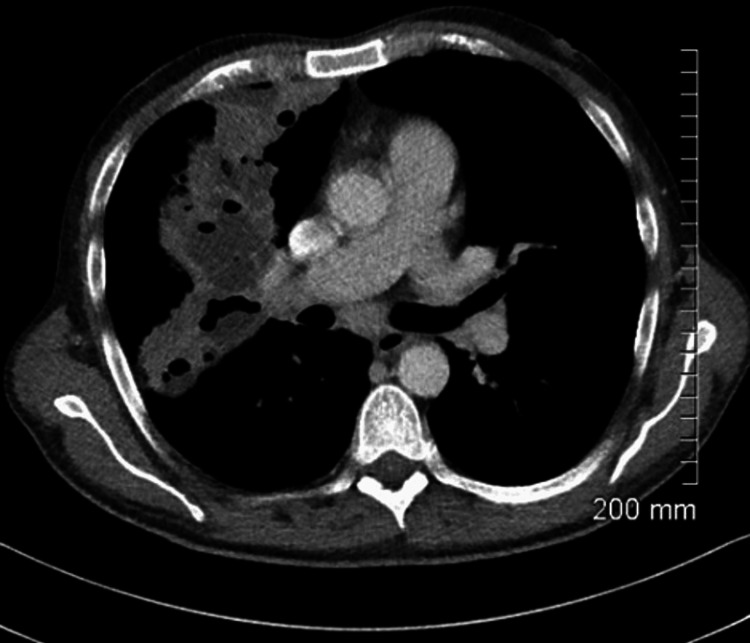
CT chest on admission

**Figure 4:**
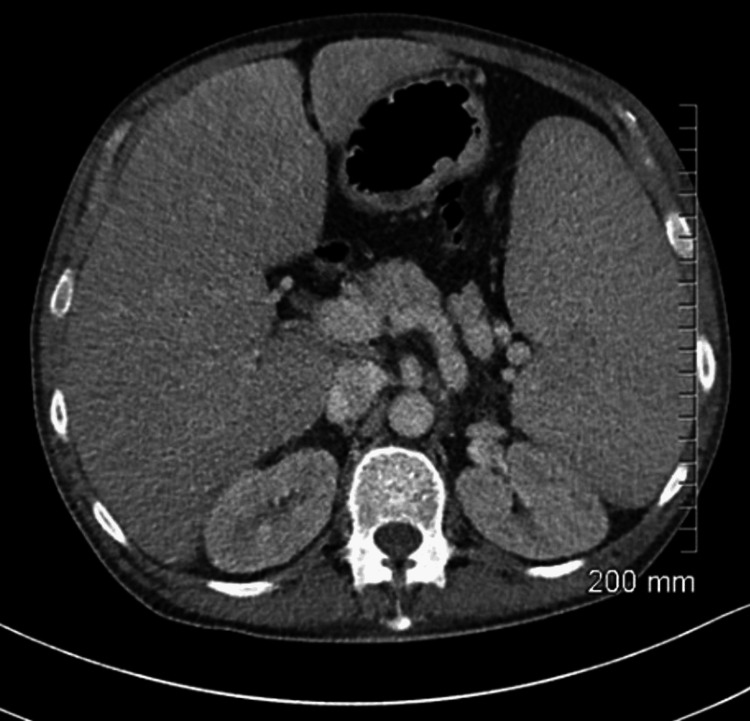
CT abdomen showing hepatosplenomegaly

On assessment in the emergency department, he was hypotensive, tachycardia, and febrile (38 °C). On examination of the patient’s chest, there were bilateral crackles and reduced air entry on the right basal and middle zones. Abdomen examination revealed moderate splenomegaly. Laboratory findings detailed pancytopenia and were suggestive of neutropenic sepsis with a high C-reactive protein (CRP) of 208, white blood cell count (WBC) of 1.5, neutrophil of 0.8, platelets of 129, and haemoglobin (HB) of 8.7. Coagulation studies showed an international normalised ratio (INR) of 1.4 (Table 1). His chest X-ray showed a right upper lobe cavitating consolidation with bulky hilar lymphadenopathy (Figure 1). Management of sepsis was commenced with oxygen 2 L, intravenous (IV) piperacillin/tazobactam 4 g/0.5 g, and IV fluid. A full workup for pancytopenic sepsis was initiated. Liver function tests (LFTs) were impaired. A blood film showed a left shift with atypical lymphocytes. The fibrinogen level was normal (4 g/L), and fibrinogen degradation products (FDPs) were also normal. CT thorax, abdomen, and pelvis showed a confluent intrapulmonary lesion suggestive of lung abscesses, hepatosplenomegaly, and mild ascites (Figures 2-4).

**Table 1:** Lab findings on admission and discharge ^1^CRP = C-reactive protein, HB = Haemoglobin, WBC = White blood cell, INR = International normalised ratio, ESR = Erythrocyte sedimentation rate, Anti-CCP = Anti-cyclic citrullinated peptide, ALT = Alanine transaminase, ALP = Alkaline phosphatase

Parameter^1^	On admission	On discharge
CRP	258 mg/L (<5)	12.5mg/L (<5)
HB	87 g/L (130-180)	139 g/L (130-180)
WBC	1.3x10^9/L (3.6-11.0)	4.6x10^9/L (3.6-11.0)
neutrophil	0.69x10^9/L (1.8-7.5)	2.47x10^9/L (1.8-7.5)
Platelets	138x10^9/L (140-400)	213x10^9/L (140-400)
Urea	15.8 mmol/L (2.5-7.0)	3.6 mmol/L (2.5-7.0)
Creatinine	177 umol/L (50-120)	70 umol/L (50-120)
INR	1.4	1
ESR	131 mm/h (1-7)	35 mm/h (1-7)
Anti-CCP	Positive	
ALT	50 U/L (1-40)	17 U/L (1-40)
Total Bilirubin	25.1 µmol/L (1-21)	7.1 µmol/L (1-21)
ALP	310 U/L (30-130)	100 U/L (30-130)
Albumin	16 g/L (35-50)	36 g/L (35-50)
Bone marrow aspirate and trephine biopsy	Hypercellular reactive features no evidence of overt dysplasia, increased immature mononuclear cells, and left shift.	

**Figure 5:**
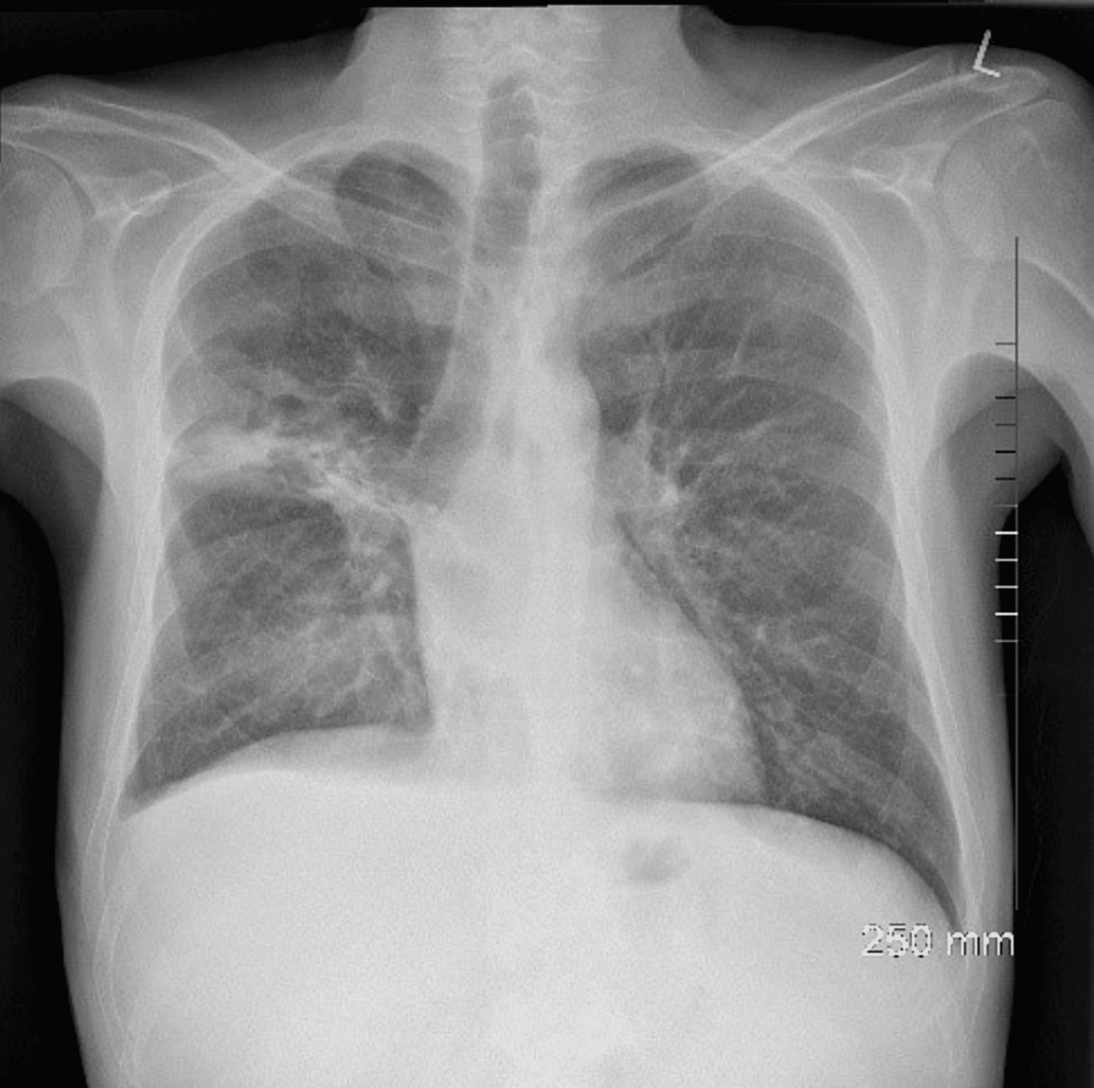
Chest X-ray post treatment

**Figure 6:**
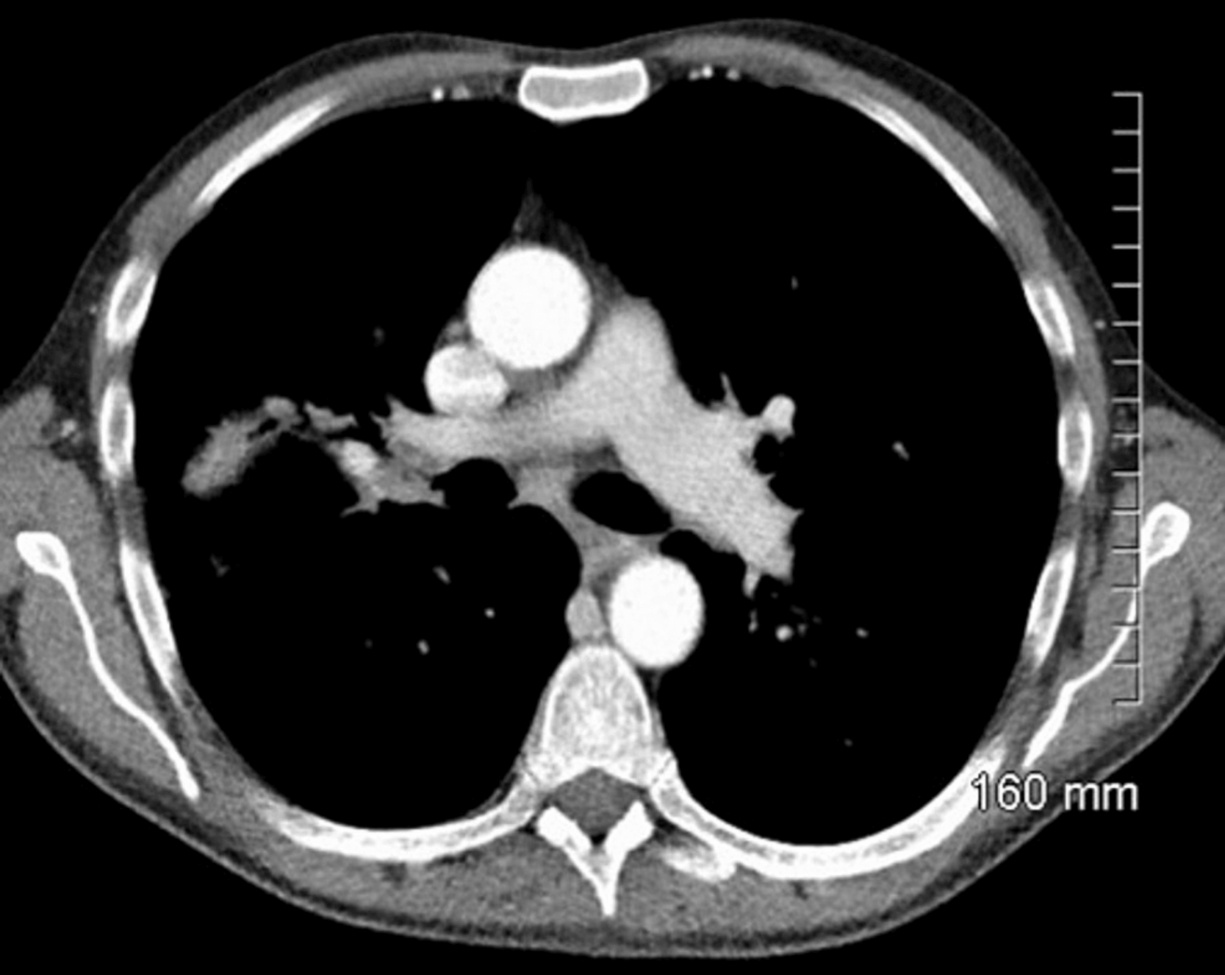
CT post treatment

The patient's clinical condition slightly improved with antibiotics, and his oxygen demands decreased, allowing cessation of supplemental oxygen. The patient had multiple temperature spikes, despite no growth on serial blood cultures. Virology screen was negative, and sputum culture was also negative, including for tuberculosis. Echocardiography was unremarkable. Endobronchial ultrasound showed thick secretions, otherwise unremarkable. Candida albicans was detected on the broncho-alveolar lavage. Accordingly, he was commenced on systemic antifungal (anidulafungin) medication. Subsequently, the patient improved clinically with no more temperature spikes, and CRP normalised. A repeat follow-up chest X-ray (Figure 5) CT thorax, abdomen, and pelvis (Figure 6) showed improvement in the lung lesion and resolution of the ascites. Vasculitis screen results reported significantly high RF of > 650 and ESR of 131, and antinuclear antibody immunoglobulin G (ANA IgG) was positive. Complement C3 and C4 levels were normal, and anti-extractable nuclear antigen (anti-ENA) and anti-(double-stranded)-DNA panel were negative.

**Figure 7:**
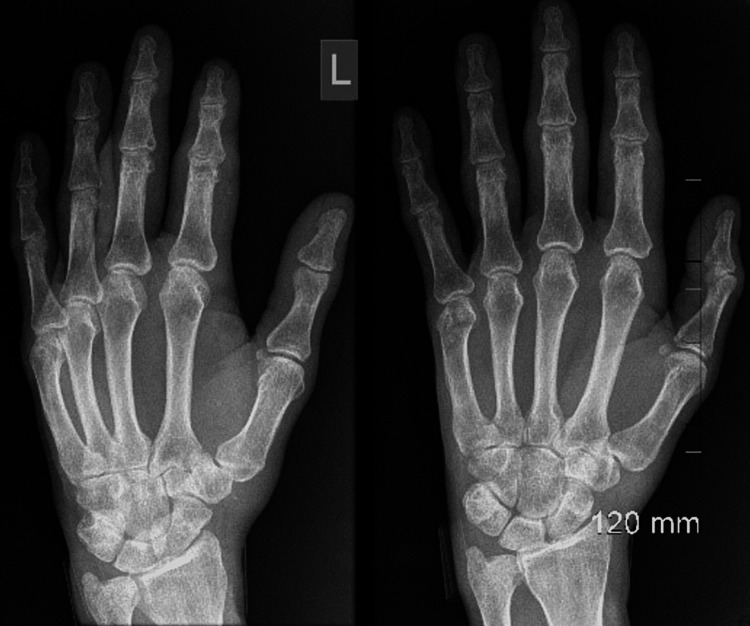
Left hand X-ray during rheumatoid flare-up

The patient had a rheumatoid flare-up during the course of his admission with left-hand swelling; thus, X-ray was done, and there was no evidence of erosive disease (Figure 7). The decision was made to commence oral steroids after resolution of the infection, which dramatically improved the clinical and haematological picture; neutrophils increased to 4, HB to 11, and platelets to > 150.

## Discussion

FS is a late manifestation of severe active RA; hence, a high index of suspicion and awareness is needed in patients presenting with neutropaenia and splenomegaly with no identifiable cause.

While FS develops after an average of 16 years [3], this patient was diagnosed with RA only two years prior to presentation with a Candida albicans lung abscess secondary to FS. This case showed a dramatic response to systemic antifungals, antibiotics, and corticosteroids with clinical and radiological resolution of the abscess and regression of the spleen size. A short course of corticosteroids was offered to control the flare-up of RA while inpatient as the patient opted not to start DMARD or biologics. The incidence of FS is reducing due to the increased use of methotrexate and biologic therapy [1]. However, in this case, the patient was not on DMARDs or biologics therapy, which might explain the early development of FS.

Unusual infections, including lung abscesses, are commonly reported complications of long-standing RA with a full recovery [6]. LGL leukaemia must also be considered, and 30%-40% of FS cases may have LGL leukaemia expansions [5]. LGL was ruled out in this case.

The main limitation of this report is the lack of data on the initial presenting features of RA two years prior to the development of FS (pattern of arthritis, serology, and radiology workup).

## Conclusions

This case details an unusual presentation of FS in a patient with a two-year duration of RA diagnosis; there was also no active arthritis at the time of admission. This emphasises the importance of considering FS in the workup for any patient presenting with neutropenia and unusual infections even in the absence of articular involvement.
